# Skin cancer risk of menopausal hormone therapy in a Korean cohort

**DOI:** 10.1038/s41598-023-37687-9

**Published:** 2023-06-29

**Authors:** Jin-Sung Yuk, Soo-Kyung Lee, Ji An Uh, Yong-Soo Seo, Myounghwan Kim, Myoung Shin Kim

**Affiliations:** 1 Department of Obstetrics and Gynaecology, School of Medicine, Sanggye Paik Hospital, Inje University, Seoul, Republic of Korea; 2 Department of Dermatology, School of Medicine, Sanggye Paik Hospital, Inje University, 1342, Dongil-Ro, Nowon-Gu, Seoul, 01757 Republic of Korea

**Keywords:** Cancer, Skin diseases, Epidemiology, Oncology, Risk factors

## Abstract

Conflicting studies exist on the association between menopausal hormone therapy (MHT) and skin cancers, such as melanoma and non-melanoma skin cancer (NMSC). This retrospective cohort study aimed to evaluate the risk of skin cancer from MHT using data from 2002 to 2019 from the National Health Insurance Service in South Korea. We included 192,202 patients with MHT and 494,343 healthy controls. Women > 40 years who had menopause between 2002 and 2011 were included. Patients with MHT had at least one MHT for at least 6 months and healthy controls had never been prescribed MHT agents. We measured the incidence of melanoma and NMSC. Melanoma developed in 70 (0.03%) patients with MHT and 249 (0.05%) controls, while the incidence of NMSC was 417 (0.22%) in the MHT group and 1680 (0.34%) in the controls*.* Tibolone (hazard ratio [HR] 0.812, 95% confidence interval [CI] 0.694–0.949) and combined oestrogen plus progestin by the manufacturer (COPM; HR 0.777, 95% CI 0.63–0.962) lowered the risk of NMSC, while other hormone groups did not change the risk. Overall, MHT was not associated with melanoma incidence in menopausal Korean women. Instead, tibolone and COPM were associated with a decrease in NMSC occurrence.

## Introduction

Hormones can affect the development of skin tumors, and this is supported by reports of changes in nevi and melanoma during pregnancy, such as their enlargement and darkening. Melanocytes are known to respond to oestrogen stimulation, and in vitro studies have found that skin treated with oestrogen can express up to three times the amount of melanin^1^. Melanoma is also reported as the most frequent cancer diagnosed during pregnancy, and studies suggest that prolonged oestrogen exposure may affect melanoma risk^2,3^. With regards to keratinocyte carcinomas (KCs), oestrogens and progestins have been proposed to act as keratinocyte proliferative factors and/or photosensitizing agents^4^. However, the results published to date are insufficient to elucidate their roles. Menopausal hormone therapy (MHT) is known to increase the risk of certain cancers, such as breast cancer^5^, but evidence for an association with skin cancer has shown conflicting results. Tang et al.^6^ reported that MHT did not affect the incidence of melanoma and non-melanoma skin cancer (NMSC), both for conjugated equine oestrogen (CEE) plus medroxyprogesterone acetate (MPA) therapy and CEE-only therapy in randomised trials of the Women’s Health Initiative (WHI). In contrast, a case–control study showed a dose-dependent increased risk of melanoma with oestrogen use^7^. Cahoon et al.^8^ reported that MHT was associated with an increased risk of basal cell carcinoma, the most common NMSC. However, there are several caveats when analysing the results of previous studies. First, some studies included the results of the use of CEE-MPA, which has been reported to increase the risk of breast cancer^9^. Second, the Women’s Health Initiative study included relatively older participants with an average age of 63 years, and approximately two-thirds of them had menopause for longer than a decade. In addition, the proportion of overweight or obese women is much higher (69.5%) than that of normal or underweight women^10^. Therefore, we aimed to evaluate the risk of skin cancer associated with MHT by using Korea's national health insurance data.

## Methods

### Database

This retrospective cohort study used data from the National Health Insurance Service (NHIS) from 01 January 2002 to 31 December 2019. Around 98% of the population is eligible to receive healthcare coverage under the NHIS in South Korea^11^. The database includes health insurance information (age, sex, diagnosis, treatment, prescription, health insurance percentile) for most patients covered by the NHIS during the period of interest. In particular, a cancer diagnosis can be registered only after pathologic confirmation, so that claims data from the NHIS, especially cancer diagnoses, are considered reliable^12^.

The NHIS covers national screening, including cardiovascular examinations, for all employees, insured individuals aged 40 or more every other year, and for blue-collar employees every year^13^. The National Cancer Screening Programme (NCSP) provides free screening for stomach, colorectal, breast, and cervical cancer to all individuals based on their age and sex. Liver and lung cancer screening examination is provided to all individuals with high-risk factors^14^. Through the NCSP, the NHIS gathers cancer screening results with additional self-administered questionnaire data, including past medical history.

Diagnoses were recorded in the database using the International Classification of Diseases, 10th revision (ICD-10). Surgeries and procedures were recorded using the Korea Health Insurance Medical Care Expenses (2012, 2016, 2019 version).

### Selection of participants

Women over the age of 40 who had menopause between 2002 and 2011 were selected as cases and controls. The MHT group included women who had used at least one MHT for at least 6 months between 2002 and 2011, whereas the non-MHT group included those who had never been prescribed MHT agents between 2002 and 2019.

We excluded the following patients from both groups: (1) those who documented menopause before 2003 (wash-out); (2) those who documented menopause before the age of 40 years in the questionnaire; and (3) those with a diagnosis code of any kind of cancer (C code) or skin diseases (L1, L4–5, L8–9) before 180 days had elapsed from the study inclusion date.

Melanoma and NMSC were defined as ICD-10 codes C43 and C44, respectively. Melanoma was defined as three or more visits to hospitals with a diagnosis code of C43. NMSC was defined as three or more visits with a diagnosis code of C44. In the case of more than one cancer, it was defined as a total skin cancer.

### Variables

The MHT agents investigated in this study were limited to tibolone, combined oestrogen and progestin by the manufacturer (COPM), oestrogen, combined oestrogen and progestin by the physician (COPP), and topical oestrogen. A detailed list of the medications is provided in Supplementary Table S1. If two or more MHT agents were used sequentially, they were assigned to the last MHT group used for > 6 months.

Age, body mass index (BMI), socioeconomic status (SES), region, Charlson Comorbidity Index (CCI), parity, age at menarche, age at menopause, smoking, alcohol consumption, physical exercise, and the period from menopause to inclusion were investigated. The reference date was the registration date of the participants in this study. Obesity was defined by the Asia–Pacific classification^15^. Low SES was defined as the case in which medical aid was applied as medical insurance, and if the administrative district of the residence was a metropolitan city, it was defined as an urban area. The CCI was calculated from one year before the date of participation to the date of participation in the study, using the names of diagnoses at medical institutions^16^. Smoking history was classified as never, past, and current. Alcohol history was classified according to the number of drinks per week. Physical exercise was classified according to the frequency of exercise for 30 min or more per week.

### Statistical analyses

All statistical tests were two-sided, and p-values less than 0.05 were considered statistically significant. Continuous variables are expressed as median [25th–75th percentile], and categorical variables are expressed as numbers or percentages. To evaluate the association between MHT use and skin cancer risk, we used the Cox proportional hazards model. To ensure the robustness of this study, only cases prescribed by obstetricians or gynaecologists were analysed for the MHT group. We used the pairwise deletion method to handle missing values. The start date of the MHT group was defined as the date on which the first MHT was prescribed, whereas that of the control group was defined as the day on which the national health and cancer check-up was performed. If only the examination year was recorded, June 30 of the health examination year was defined as the starting date. In the absence of special events, the last day of the study was set as the date of death or 31 December 2019. All statistical analyses were conducted using the SAS Enterprise Guide 6.1 (SAS Institute, Cary, NC, USA).

### Ethics

This study was approved by the institutional review board of Inje University Sanggye Paik Hospital (SGPAIK-2020-08-002). We were able to export only the de-identified data relevant to the study from the virtual server within the NHIS using the NHIS privacy policy. The requirement for informed consent from the study subjects was waived by the institutional review board of Inje University Sanggye Paik Hospital because study would not affect rights and welfare of subject and could not be practicably carried out without the waiver. This study was conducted in accordance with the principles of the Declaration of Helsinki.

## Results

### Demographic characteristics of participants

**Figure 1:**
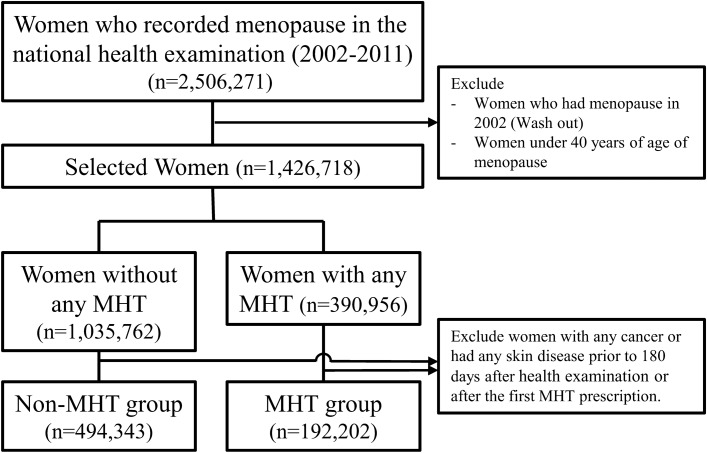
Flowchart to select case–control according to MHT in Korea National Health Insurance Data (2002–2019). *MHT* Menopausal hormone therapy.

Among the 2,506,271 women who reported menopause during the medical examination from 2002 to 2011, 192,202 women were included in the MHT group, and 494,343 women were included in the non-MHT group (Fig. 1). The average age of the participants was 56 [52–62] years old. Table 1 and Supplementary Table S2 show the detailed characteristics of the participants in this study. The average duration of hormone therapy was 22 [10–54] months (Table 2).

**Table 1:** Characteristics of women according to menopausal hormone exposure status at recruitment, Korea National Health Insurance Data, from 2002 to 2019.

	Non-MHT	Tibolone	CEPM	Oral estrogen	CEPP	Topical estrogen	Total
Number of women	494,343	97,074	60,776	29,478	3686	1188	686,545
Median age (years)	58 [52–64]	54 [50–58]	52 [50–56]	52 [49–57]	54 [51–59]	53 [50–57]	56 [52–62]
Age at inclusion (years)							
40–49	48,869 (9.9)	17,283 (17.8)	14,132 (23.3)	7882 (26.7)	665 (18)	265 (22.3)	89,096 (13)
50–59	236,883 (47.9)	62,392 (64.3)	39,636 (65.2)	16,151 (54.8)	2156 (58.5)	715 (60.2)	357,933 (52.1)
60–69	150,340 (34.5)	15,583 (16.4)	6457 (10.7)	4524 (15.8)	756 (21.1)	186 (16)	177,846 (28.5)
≥ 70	58,251 (11.8)	1816 (1.9)	551 (0.9)	921 (3.1)	109 (3)	22 (1.9)	61,670 (9)
Median BMI (kg/m^2^)	23.9 [22.1–26]	23.5 [21.8–25.4]	23.2 [21.5–25.1]	23.8 [22–25.8]	23.4 [21.6–25.2]	23.8 [22.1–25.7]	23.8 [21.9–25.8]
BMI (kg/m^2^)							
< 18.5	9560 (2)	1726 (1.8)	1229 (2)	451 (1.5)	74 (2)	22 (1.9)	13,062 (1.9)
18.5–22.9	167,506 (34.7)	38,181 (39.8)	26,883 (44.6)	10,628 (36.4)	1528 (41.9)	436 (37)	245,162 (36.4)
23–24.9	127,678 (26.4)	26,754 (27.9)	16,224 (26.9)	8148 (27.9)	1011 (27.7)	315 (26.8)	180,130 (26.7)
25–29.9	158,267 (32.7)	26,853 (28)	14,731 (24.4)	8939 (30.6)	953 (26.1)	372 (31.6)	210,115 (31.2)
≥ 30	20,303 (4.2)	2435 (2.5)	1204 (2)	1017 (3.5)	83 (2.3)	32 (2.7)	25,074 (3.7)
SES							
Mid-high SES	472,042 (95.5)	93,364 (96.2)	59,144 (97.3)	28,599 (97)	3600 (97.7)	1152 (97)	657,901 (95.8)
Low SES	22,301 (4.5)	3710 (3.8)	1632 (2.7)	879 (3)	86 (2.3)	36 (3)	28,644 (4.2)
Region							
Urban area	153,296 (31)	31,814 (32.8)	21,960 (36.1)	9751 (33.1)	1937 (52.6)	562 (47.3)	219,320 (31.9)
Rural area	341,047 (69)	65,260 (67.2)	38,816 (63.9)	19,727 (66.9)	1749 (47.4)	626 (52.7)	467,225 (68.1)
CCI							
0	331,584 (67.1)	68,555 (70.6)	44,727 (73.6)	21,214 (72)	2619 (71.1)	807 (67.9)	469,506 (68.4)
1	88,339 (17.9)	17,511 (18)	10,040 (16.5)	4925 (16.7)	632 (17.1)	184 (15.5)	121,631 (17.7)
≥ 2	74,420 (15.1)	11,008 (11.3)	6009 (9.9)	3339 (11.3)	435 (11.8)	197 (16.6)	95,408 (13.9)
Smoking							
Never	450,318 (96.6)	87,386 (94)	54,842 (93.7)	26,805 (94.9)	3395 (95.8)	1090 (96.2)	623,836 (95.9)
Past	4497 (1)	1494 (1.6)	1036 (1.8)	379 (1.3)	46 (1.3)	13 (1.1)	7465 (1.1)
Current	11,531 (2.5)	4092 (4.4)	2664 (4.6)	1056 (3.7)	104 (2.9)	30 (2.6)	19,477 (3)
Alcohol (per week)							
None	401,256 (85.6)	73,285 (78.2)	45,263 (76.8)	22,953 (80.6)	2984 (83.6)	943 (82.1)	546,684 (83.5)
≤ 2/week	58,354 (12.5)	17,536 (18.7)	11,787 (20)	4847 (17)	514 (14.4)	187 (16.3)	93,225 (14.2)
3–6/week	6429 (1.4)	2143 (2.3)	1507 (2.6)	485 (1.7)	53 (1.5)	15 (1.3)	10,632 (1.6)
Daily	2593 (0.6)	730 (0.8)	395 (0.7)	208 (0.7)	17 (0.5)	3 (0.3)	3946 (0.6)
Physical exercise (per week)							
None	301,412 (64.2)	54,951 (58.7)	34,883 (59.2)	16,854 (59.2)	2031 (56.9)	601 (52.7)	410,732 (62.7)
1–2	80,022 (17.1)	18,166 (19.4)	11,710 (19.9)	5509 (19.4)	715 (20)	246 (21.6)	116,368 (17.8)
3–4	43,862 (9.3)	10,766 (11.5)	6851 (11.6)	3127 (11)	442 (12.4)	166 (14.5)	65,214 (10)
5–6	14,004 (3)	3514 (3.8)	2208 (3.7)	970 (3.4)	143 (4)	49 (4.3)	20,888 (3.2)
Daily	29,993 (6.4)	6243 (6.7)	3308 (5.6)	2008 (7.1)	239 (6.7)	79 (6.9)	41,870 (6.4)

*BMI* Body mass index, *CCI* Charlson comorbidity index, *COPM* combined oestrogen plus progestin by manufacturer, *COPP* combined oestrogen plus progestin by physician, *MHT* menopausal hormone therapy, *SES* socioeconomic status.

Gynaecological characteristics of women according to menopausal hormone exposure are presented in Supplementary Table S2.

Data are expressed as the number (%) or median [25 percentile, 75 percentile].

**Table 2:** Characteristics of women with menopausal hormone therapy, Korea National Health Insurance Data, from 2002 to 2019.

MHT characteristics	Tibolone	Combined oestrogen plus progestin by manufacturer	Oral oestrogen	Combined oestrogen plus progestin by physician	Topical oestrogen	Total MHT
Median duration (months)	25 [11–58]	24 [11–56]	15 [8–38]	16 [9–33]	14 [8–27]	22 [10–54]
Duration (years)						
< 5	73,258 (75.5)	46,609 (76.7)	24,593 (83.4)	3225 (87.5)	1122 (94.4)	148,807 (77.4)
5–9.9	16,741 (17.2)	10,403 (17.1)	3226 (10.9)	335 (9.1)	63 (5.3)	30,768 (16)
≥ 10	7075 (7.3)	3764 (6.2)	1659 (5.6)	126 (3.4)	3 (0.3)	12,627 (6.6)
Duration of previous other MHT (years)						
< 5	94,872 (97.7)	59,968 (98.7)	29,107 (98.7)	3130 (84.9)	1175 (98.9)	188,252 (97.9)
5–9.9	1949 (2)	729 (1.2)	324 (1.1)	398 (10.8)	13 (1.1)	3413 (1.8)
≥ 10	253 (0.3)	79 (0.1)	47 (0.2)	158 (4.3)	(0)	537 (0.3)
Last dosage of tibolone (per day)						
1.25 mg	888 (0.9)					
2.5 mg	96,081 (99)					
Over 5 mg	94 (0.1)					
Prescribed specialty						
Gynaecology	32,170 (33.1)	27,539 (45.3)	11,818 (40.1)	852 (23.1)	289 (24.3)	72,668 (37.8)
Non-gynaecology	64,904 (66.9)	33,237 (54.7)	17,660 (59.9)	2834 (76.9)	899 (75.7)	119,534 (62.2)

*MHT* menopausal hormone therapy.

Data are expressed as the number (%) or median [25 percentile, 75 percentile].

### Risk of melanoma and NMSC

Melanoma developed in 249 (0.05%, 249/494,343) patients in the non-MHT group and in 70 (0.03%, 70/192,202) patients in the MHT group (Table 3). In the MHT group, melanoma developed in 31 (0.03%, 31/97,074) tibolone, 21 (0.03%, 21/60,776) COPM, 14 (0.05%, 14/29,478) oral oestrogen, 3 (0.08%, 3/3686) COPP, and 1 (0.08%, 1/1188) topical oestrogen patient, respectively. The incidence of NMSC was 1680 cases (0.34%, 1680/494,343) in the non-MHT group and 417 cases (0.22%, 417/192,202) in the MHT group. In terms of hormone use, NMSC developed in 211 (0.22%, 211/97,074) tibolone, 109 (0.18%, 109/60,776) COPM, 84 (0.28%, 84/29,478) oral oestrogen, 10 (0.27%, 10/3686) COPP, and 3 (0.25%, 3/1188) topical oestrogen patients, respectively.

**Table 3:** Incidence of skin cancer according to hormone exposure status at recruitment, Korea National Health Insurance Data, from 2002 to 2019.

	Non-MHT	Tibolone	Combined oestrogen plus progestin by manufacturer	Oral oestrogen	Combined oestrogen plus progestin by physician	Topical oestrogen	Total
Median period from menopause to inclusion (years)	7 [2–14]	4 [1–8.5]	2.5 [0–6.5]	4.5 [1–9]	4.5 [1–9.5]	4.5 [1–9]	6 [2–12.5]
Melanoma							
Not present	494,094 (99.95)	97,043 (99.97)	60,755 (99.97)	29,464 (99.95)	3683 (99.92)	1187 (99.92)	686,226 (99.95)
Present	249 (0.05)	31 (0.03)	21 (0.03)	14 (0.05)	3 (0.08)	1 (0.08)	319 (0.05)
Non-melanoma skin cancer							
Not present	492,663 (99.66)	96,863 (99.78)	60,667 (99.82)	29,394 (99.72)	3676 (99.73)	1185 (99.75)	684,448 (99.69)
Present	1680 (0.34)	211 (0.22)	109 (0.18)	84 (0.28)	10 (0.27)	3 (0.25)	2097 (0.31)
Total skin cancer							
Not present	492,452 (99.62)	96,835 (99.75)	60,652 (99.8)	29,383 (99.68)	3673 (99.65)	1184 (99.66)	684,179 (99.66)
Present	1891 (0.38)	239 (0.25)	124 (0.2)	95 (0.32)	13 (0.35)	4 (0.34)	2366 (0.34)

*MHT* menopausal hormone therapy.

Data are expressed as the number (%) or median [25 percentile, 75 percentile].

**Figure 2:**
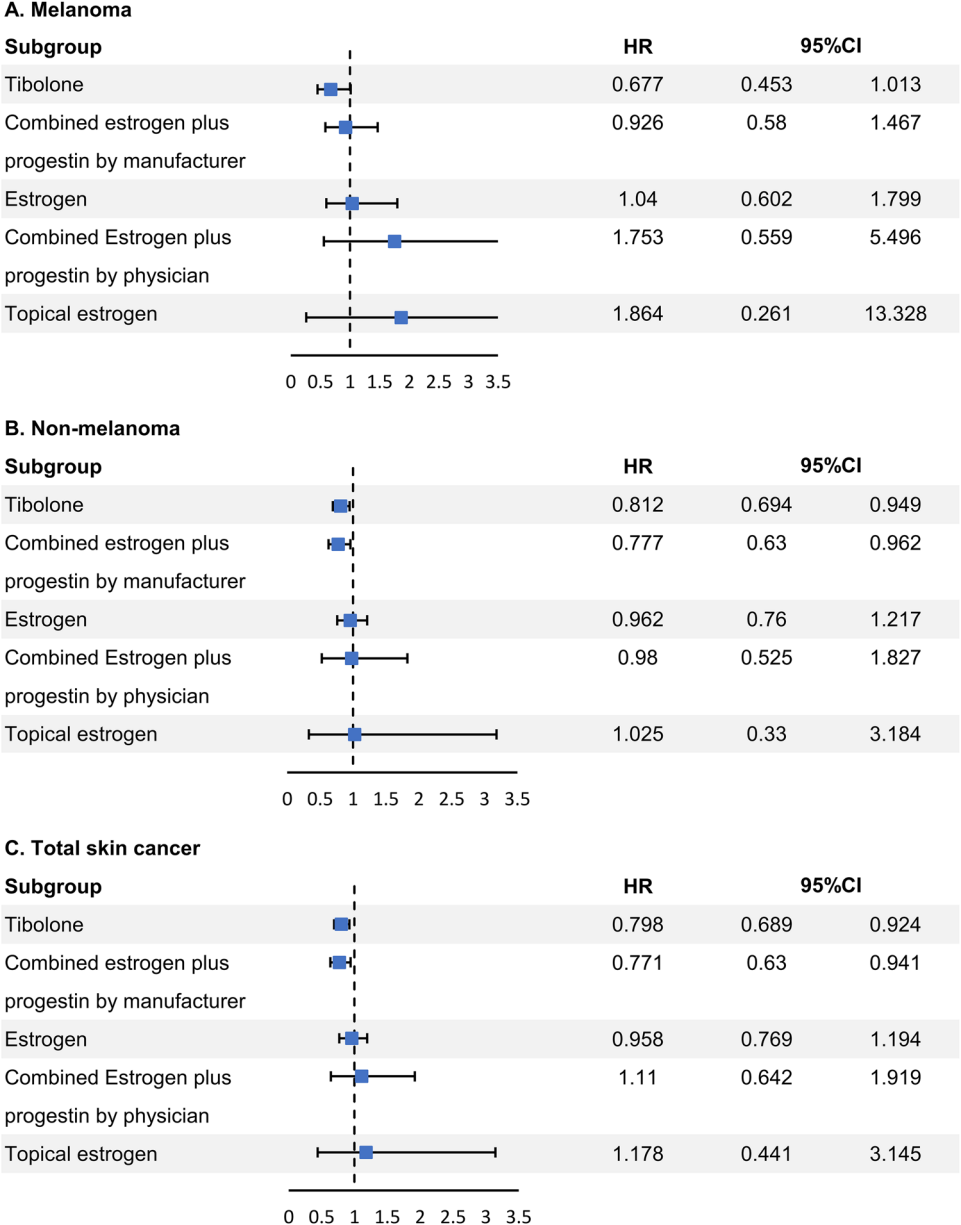
Comparison of hazards of Melanoma, NMSC, and total skin cancer development among MHT subgroup. *CI* Confidence interval, *HR* Hazard ratio, *MHT* menopausal hormone therapy, *NMSC* non-melanoma skin cancer.

MHT did not affect the incidence of melanoma in the Cox proportional regression analysis after adjusting for multiple variables (Fig. 2). We found that tibolone (hazard ratio [HR] 0.812, 95% confidence interval [CI] 0.694–0.949) and COPM (HR 0.777, 95% CI 0.63–0.962) lowered the risk of NMSC. In terms of total skin cancer, tibolone (HR 0.798, 95% CI 0.689–0.924) and COPM (HR 0.771, 95% CI 0.63–0.941) lowered the risk, while other hormone groups did not change the risk. In a subgroup analysis of tibolone only, the risk of total skin cancer did not change at 1.25 mg (half dose) of tibolone (HR 1.499, 95% CI 0.561–4.001) (Supplementary Table S3).

In the Cox proportional regression analysis, older age (≥ 70 years: HR 2.566, 95% CI 1.187–5.547) and obesity (BMI ≥ 30: HR 1.854, 95% CI 1.108–3.1) increased the risk of melanoma (Table 4). In contrast, older age (≥ 70 years: HR 5.202, 95% CI 3.767–7.183), rural areas (HR 1.272, 95% CI 1.144–1.415), age at menopause (≥ 55 years: HR 1.346, 95% CI 1.062–1.707), and long period from menopause to inclusion (≥ 10 years: HR 1.655, 95% CI 1.353–2.025) increased the risk of NMSC. However, obesity (BMI ≥ 30: HR 0.766, 95% CI 0.597–0.984), past smoking (HR 0.467, 95% CI 0.233–0.936), alcohol consumption (≤ 2/week) (HR 0.831, 95% CI 0.706–0.979), and physical exercise (1–2/week) (HR 0.805, 95% CI 0.702–0.922) lowered the risk of NMSC.

**Table 4:** Hazard ratios for risk of skin cancer according to major variables, Korea National Health Insurance Data, from 2002 to 2019.

Variables	Melanoma^a^		Non-melanoma^a^		Total skin cancer^a^	
	HR (95% CI)^a^	P-value	HR (95% CI)^a^	P-value	HR (95% CI)^a^	P-value
MHT						
Tibolone	0.677 (0.453–1.013)	0.058	0.812 (0.694–0.949)	0.009	0.798 (0.689–0.924)	0.003
CEPM	0.926 (0.584–1.467)	0.74	0.777 (0.628–0.962)	0.021	0.771 (0.633–0.941)	0.01
Oral estrogen EOOOEstrogen	1.04 (0.602–1.799)	0.89	0.962 (0.76–1.217)	0.75	0.958 (0.769–1.194)	0.71
CEPP	1.753 (0.559–5.496)	0.34	0.98 (0.525–1.827)	0.95	1.11 (0.642–1.919)	0.71
Topical estrogen	1.864 (0.261–13.328)	0.54	1.025 (0.33–3.184)	0.97	1.178 (0.441–3.145)	0.74
Age at inclusion (years)						
50–59	1.207 (0.731–1.994)	0.46	1.463 (1.159–1.848)	0.001	1.408 (1.137–1.743)	0.002
60–69	1.938 (0.979–3.839)	0.058	2.385 (1.769–3.216)	< 0.001	2.268 (1.721–2.99)	< 0.001
≥ 70	2.566 (1.187–5.547)	0.017	5.202 (3.767–7.183)	< 0.001	4.723 (3.5–6.373)	< 0.001
BMI (kg/m^2^)						
< 18.5	0.439 (0.108–1.786)	0.25	0.672 (0.46–0.981)	0.04	0.662 (0.46–0.955)	0.027
23–24.9	1.408 (1.042–1.903)	0.026	0.873 (0.777–0.981)	0.023	0.92 (0.824–1.027)	0.14
25–29.9	1.342 (1.002–1.797)	0.048	0.841 (0.753–0.939)	0.002	0.894 (0.805–0.992)	0.034
≥ 30	1.854 (1.108–3.1)	0.019	0.766 (0.597–0.984)	0.037	0.871 (0.695–1.093)	0.23
SES						
Low SES	1.363 (0.741–2.509)	0.32	1.217 (0.949–1.561)	0.12	1.232 (0.975–1.555)	0.08
Region						
Rural area	1.036 (0.806–1.331)	0.78	1.272 (1.144–1.415)	< 0.001	1.242 (1.125–1.371)	< 0.001
CCI						
1	1.072 (0.798–1.441)	0.64	1.072 (0.954–1.204)	0.24	1.053 (0.944–1.175)	0.36
≥ 2	1.138 (0.826–1.568)	0.43	1.096 (0.963–1.247)	0.17	1.091 (0.966–1.232)	0.16
Parity (years)						
0	0.961 (0.523–1.767)	0.90	1.193 (0.915–1.556)	0.19	1.128 (0.883–1.441)	0.34
2	0.94 (0.551–1.602)	0.82	1.056 (0.829–1.344)	0.66	1.03 (0.826–1.285)	0.79
≥ 3	0.877 (0.488–1.578)	0.66	1.042 (0.806–1.348)	0.75	1.007 (0.794–1.276)	0.96
Age at menarche (years)						
≥ 13	0.835 (0.566–1.234)	0.37	0.863 (0.74–1.008)	0.063	0.873 (0.755–1.01)	0.067
Age at menopause (years)						
45–49	1.058 (0.71–1.577)	0.78	1.057 (0.898–1.243)	0.50	1.079 (0.926–1.257)	0.33
50–54	1.091 (0.71–1.676)	0.69	1.224 (1.034–1.449)	0.019	1.229 (1.048–1.441)	0.011
≥ 55	0.806 (0.419–1.552)	0.52	1.346 (1.062–1.707)	0.014	1.308 (1.044–1.638)	0.02
Smoking						
Past	0.641 (0.159–2.586)	0.53	0.467 (0.233–0.936)	0.032	0.504 (0.271–0.94)	0.031
Current	0.755 (0.334–1.711)	0.50	0.991 (0.735–1.338)	0.96	0.979 (0.739–1.297)	0.88
Alcohol (g/week)						
≤ 2/week	1.173 (0.833–1.65)	0.36	0.831 (0.706–0.979)	0.026	0.872 (0.751–1.012)	0.071
3–6/week	0.854 (0.272–2.684)	0.79	0.907 (0.569–1.448)	0.68	0.915 (0.594–1.411)	0.69
Daily	0.619 (0.087–4.42)	0.63	0.643 (0.306–1.352)	0.24	0.652 (0.325–1.307)	0.23
Physical exercise (per week)						
1–2	0.817 (0.588–1.136)	0.23	0.805 (0.702–0.922)	0.002	0.821 (0.724–0.931)	0.002
3–4	0.789 (0.515–1.208)	0.28	0.786 (0.659–0.938)	0.008	0.791 (0.671–0.933)	0.005
5–6	1.044 (0.552–1.973)	0.90	0.749 (0.551–1.019)	0.066	0.78 (0.588–1.035)	0.085
Daily	0.754 (0.465–1.223)	0.25	0.867 (0.726–1.036)	0.12	0.856 (0.724–1.013)	0.071
Period from menopause to inclusion (years)						
5–9	1.249 (0.868–1.796)	0.23	1.169 (0.995–1.373)	0.057	1.184 (1.02–1.373)	0.026
≥ 10	1.321 (0.807–2.161)	0.27	1.655 (1.353–2.025)	< 0.001	1.613 (1.336–1.947)	< 0.001

*BMI* Body mass index, *CCI* Charlson comorbidity index, *CEPM* combined oestrogen and progestin by the manufacturer, *CEPP* combined oestrogen and progestin by the physician (COPP), *CI* confidence interval, *HR* hazard ratio, *MHT* menopausal hormone therapy, *SES* socioeconomic status.

^a^HRs were adjusted for age group, body mass index, socioeconomic status, region, Charlson comorbidity index, parity, age at menarche, age at menopause, smoking, alcohol, physical exercise, period from menopause to inclusion.

## Discussion

In this large, population-based, retrospective study, we found that MHT did not affect the incidence of melanoma in menopausal Korean women, and we did not find an association between oral oestrogen and melanoma or NMSC. Our findings are consistent with the results of the WHI trial^6^, which showed that MHT did not affect the overall incidence of NMSC or melanoma in menopausal women in the US. Notably, we observed a decreased risk for NMSC with tibolone or COPM in this population. To our knowledge, these results are the first large-scale data on tibolone and COPM and the risk of NMSC or melanoma.

In recent years, there have been only a limited number of studies investigating the association between MHT and NMSC, with the majority of them focusing on Caucasian populations. While Tang et al.^6^ found no correlation between MHT use and the risk of NMSC, most studies have reported an increased risk of NMSC with MHT use^17^. For instance, a Danish study^18^ reported a 15% increase in the risk of basal cell carcinoma (BCC) among MHT ever-users, while a US study^8^ analysing 1730 BCC cases found similar results, suggesting a possible link between MHT and NMSC, especially BCC. A study by Kuklinski et al.^19^ reported that MHT users had more aggressive BCC and suggested that oestrogen receptor activation induced proliferation and increased susceptibility to environmental insults. However, the same study reported no association between MHT use and the incidence of BCC. Our finding that tibolone and COPM lower the risk of NMSC is distinct from those of previous studies. Differences in these results may be due to racial or ethnic differences or because previous studies included analyses only for unopposed oestrogen and oestrogen/progestin, but not tibolone and COPM. It is worth noting that both tibolone and COPM have progestogenic activity or contain progesterone. Oestrogens and progestins are known as photosensitizing agents that induce proliferation of keratinocytes, and are crucial modulators of epidermal carcinogenesis^20^. Keratinocytes express both oestrogen receptors (ER)-α and ER-β, and at physiological concentrations, oestradiol only increases the expression of ER-α receptors^21^. Isolated upregulation of ER-α induced by oestrogen may reduce the relative effects of ER-β, which is an important tumor suppressor, thus explaining the relationship between oestrogen and epidermal carcinogenesis. Conversely, a previous study showed that progesterone downregulated Wnt/β-catenin signalling^22^, which is known as a significant pathway in the development of BCC and SCC^23^. Based on these observations, we propose that progestins may play beneficial roles in the development of NMSC. Interestingly, our study found that COPM had a protective effect on NMSC, while COPP did not show a significant association with NMSC. This difference in results could be attributed to the specific properties of each progesterone drug. Another possibility is that the lower incidence of NMSC in the COPP group was not statistically significant due to the small number of patients in that group.

We analysed parity, age at menarche, and age at menopause, and these variables indirectly measure physiological exposure to sex hormones. These factors were not associated with the risk of melanoma; however, age at menopause (≥ 55 years) was associated with an increased risk of NMSC. Similar to our results, a Finnish study found that the risk of BCC decreased with an increasing number of deliveries^24^.

Additionally, our results unexpectedly showed that obesity and smoking history, which are commonly known risk factors for cancer, lowered the risk of NMSC. Karimi et al.^25^ reviewed previous studies and reported that obesity is closely associated with an increase in melanoma and NMSC. The associations between obesity and skin cancer are not fully understood, but it is anticipated that obesity-associated inflammation, oxidative stress, hormones, and metabolic pathways are involved^25^. In our study, obese menopausal women had a lower risk of NMSC. Our result supports a previous study in which obesity lowered the risk of NMSC, which was attributed to less outdoor activity and less chronic sun exposure in obese people^26^.

Generally, smoking is considered to increase the risk of skin cancer^27^; however, evidence has not yet reached a definite conclusion. A meta-analysis of cohort studies on smoking and skin cancer reported that current smoking was associated with SCC, but inversely associated with BCC and melanoma^27^. They also reported that smoking was not associated with the risk of skin cancer^27^. Although SCC and BCC were not analysed separately in our study, our results are similar to those of the aforementioned study. We observed that smoking was not associated with an increased risk of melanoma and NMSC, whereas past smoking was associated with a decreased risk of NMSC. The underlying mechanism of the beneficial effects of smoking or past smoking on NMSC was inconclusive, but one of the hypotheses suggested that smoking might decrease BCC risk by interacting with genes conferring susceptibility to BCC^27^. Further studies are required to clarify the association between these factors and skin cancer in menopausal women.

This study has several strengths. A large sample size gives this study statistical power compared to other observational studies, and because it is based on nationwide registry data, it was possible to increase the accuracy of cancer incidence and minimise selection bias. This was an observational study using tibolone as the main MHT. To the best of our knowledge, there have been no previous studies on NMSC and tibolone. Considering that tibolone is the most commonly prescribed MTH drug in South Korea, our results have an implication in real practice. Finally, variables including age, BMI, SES, region, CCI, parity, the age at menarche, age at menopause, smoking, alcohol, physical exercise, and the period from menopause to inclusion were adjusted to control confounding.

Our study has several principal limitations, the first of which pertains to the use of health insurance data and the inherent risk of overestimation this carries. In Korea, C codes are allocated based on pathological confirmation, however, this does not unequivocally ensure the completeness of the data. To address this concern, we included in our analysis only those patients who had been diagnosed with skin cancer and had made three or more visits to medical institutions for their condition. Additionally, we acknowledge the absence of specific data on alcohol consumption levels and quantities of smoking. Given that the survey questions relating to these factors varied throughout the study period (2002–2011), we concentrated on consistently reported parameters: namely, the frequency of alcohol intake and the prevalence of smoking. While this focus may constrain the comprehensiveness of our findings with regards to these behaviours, we assert that our analysis continues to yield significant insights despite these limitations. Another key limitation of this study is that our data did not contain well-known risk factors for skin cancer, such as ultraviolet (UV) exposure, family history, and immune status. In Korea, the most common type of melanoma is acral lentiginous melanoma, followed by nonchronic sun damage-induced melanoma^28^. Only a small percentage of melanoma cases (about 10%) in Korea were found in chronic sun-damaged areas^28^, suggesting a lower impact of UV exposure on melanoma risk than in the Western population. The high incidence of NMSC in chronically sun-exposed areas in Korea suggests that UV exposure is a more significant risk factor for NMSC than melanoma in this population. Moreover, this study indirectly inferred UV exposure by analysing living areas (rural/urban), finding that rural living increased the risk of developing NMSC but not melanoma (Table 4). These observations lead to the assumption that NMSC in Korea is more influenced by UV exposure than melanoma, so the limitation on UV exposure data might affect our results, especially on NMSC. Although detailed information such as chronic UV exposure, sunburn history, or sunscreen use could not be collected, we presume that UV exposure was not remarkably different after adjusting regions between the non-MHT and MHT or each MHT group because the Korean population showed relatively homogeneous ethnicity and low sunburn potential based on their Fitzpatrick skin phototypes^29^. Another limitation was that information on the subtypes of NMSC, such as BCC and SCC, could not be obtained. Considering that some previous studies showed disparate outcomes according to the subtypes of NMSC^8,18^, further evaluation of the subtypes is warranted for a better understanding of the results. Lastly, we could not obtain a detailed drug list constituting the COPM group due to the NHIS policy.

In conclusion, our study supports that MHT is not associated with melanoma in menopausal women. We propose that tibolone and combined oestrogen and progestin by the manufacturer may contribute to lowering the incidence of non-melanoma skin cancer in the Korean population. Further prospective studies involving multi-ethnic groups are needed to expand the results of this study.

## Supplementary Information

Supplementary Tables.

## Data Availability

The data that support the findings of this study are available from the corresponding author, M.S. Kim, upon reasonable request.
