# Supplementary material for: Skin cancer risk of menopausal hormone therapy in a Korean cohort
Source: Sci Rep. 2023 Jun 29;13:10572. doi: 10.1038/s41598-023-37687-9 (PMC10310700; doi:10.1038/s41598-023-37687-9)
Supplement: Supplementary file 1 — Supplementary Tables. [file 41598_2023_37687_MOESM1_ESM.pdf]

Supplementary information

Table S1. Menopausal hormones used in clinical practice by group.

MHT (generic name)	Brand name
Tibolone	
Tibolone 2.5 mg	Livial, Rabilone, Libolone, Libron, Live on, Liviem, Tiborisi, Tibolan, Tibiol, Pharmbio Korea Tibolone, Hyundai Tibolone
Combined Estrogen plus progestin by manufacturer (CEPM)	
Estradiol Hemihydrate 1.03 mg, Drospirenone 2 mg	Angeliq
Estradiol Valerate 2 mg, Medroxyprogesterone Acetate 10 mg	Divina
Estradiol Valerate 1 mg, Medroxyprogesterone Acetate 2.5 mg	Indivina Tab 1 mg/2.5 mg
Estradiol Valerate 1 mg, Medroxyprogesterone Acetate 5 mg	Indivina Tab 1 mg/5 mg
Estradiol Valerate 2 mg, Medroxyprogesterone Acetate 2.5 mg	Indivina Tab 2 mg/2.5 mg
Estradiol Hemihydrate 2 mg, Norethisterone Acetate 1 mg	Cliane
Estradiol Hemihydrate 2.06 mg, Dydrogesterone 10 mg	Femoston 2/10
Estradiol Hemihydrate 1.03 mg, Dydrogesterone 10 mg	Femoston 1/10
Estradiol Hemihydrate 1.03 mg, Dydrogesterone 5 mg	Femoston Conti
Cyproterone Acetate 1 mg, Estradiol Valerate 2 mg	Climen
Estradiol Hemihydrate 1.03 mg, Norethisterone Acetate 0.5 mg	Esdiol-half
Estradiol Valerate 1.31 mg, Norethisterone Acetate 0.5 mg	Cliovelle
Estrogen	
Conjugated Estrogens 0.3 mg	Premina 0.3 mg
Conjugated Estrogens 0.625 mg	Premina 0.625 mg
Estradiol Valerate 1 mg	Progynova 1 mg
Estradiol Valerate 2 mg	Progynova 2 mg
Estradiol Hemihydrate 1 mg	Preda 1 mg
Combined Estrogen plus progestin by physician (CEPP) [a]	
Progesterone Micronized 100 mg	Utrogestan 100 mg
Medroxyprogesterone Acetate 5 mg	Provera 5 mg
Medroxyprogesterone Acetate 10 mg	Provera 10 mg
Dydrogesterone 10 mg	Duphaston
Topical estrogen	
Estradiol Hemihydrate	Estreva Gel, Climara patch, Divigel Gel

Abbreviatnions: MHT, menopausal hormone therapy.
[a] This group used the progestin below and the estrogen group above simultaneously.

Table S2. Gynaecological characteristics of women according to menopausal hormone exposure status at recruitment, Korea National Health Insurance Data, from 2002 to 2019.

	Non-MHT	Tibolone	CEPM	Oral Estrogen	CEPP	Topical estrogen	Total
Parity (years)							
0 or not respond	108,070 (21.9)	18,187 (18.7)	9,705 (16)	6,892 (23.4)	855 (23.2)	278 (23.4)	143,987 (21)
1	27,025 (5.5)	7,895 (8.1)	5,864 (9.6)	2,127 (7.2)	237 (6.4)	87 (7.3)	43,235 (6.3)
2	283,443 (67.7)	60,935 (70)	40,103 (72)	17,237 (65.6)	2,175 (66.6)	695 (65.6)	404,588 (68.4)
≥3	75,805 (15.3)	10,057 (10.4)	5,104 (8.4)	3,222 (10.9)	419 (11.4)	128 (10.8)	94,735 (13.8)
Age at menarche (years)							
<13	98,300 (20)	15,254 (15.9)	9,285 (15.4)	5,656 (19.5)	716 (19.7)	229 (19.5)	129,440 (19)
≥13	392,532 (80)	80,879 (84.1)	51,051 (84.6)	23,401 (80.5)	2,927 (80.3)	943 (80.5)	551,733 (81)
Age at menopause (years)							
40-44	71,780 (14.5)	12,640 (13)	7,702 (12.7)	6,471 (22)	514 (13.9)	253 (21.3)	99,360 (14.5)
45-49	145,220 (29.4)	32,394 (33.4)	20,655 (34)	10,518 (35.7)	1,219 (33.1)	428 (36)	210,434 (30.7)
50-54	237,099 (52.2)	44,722 (49.8)	28,247 (49.9)	11,042 (39.4)	1,671 (49.1)	438 (39.1)	323,219 (51.1)
≥55	40,244 (8.1)	7,318 (7.5)	4,172 (6.9)	1,447 (4.9)	282 (7.7)	69 (5.8)	53,532 (7.8)
Period from menopause to inclusion (years)							
<5	188,853 (38.2)	54,217 (55.9)	39,806 (65.5)	15,308 (51.9)	1,854 (50.3)	619 (52.1)	300,657 (43.8)
5-9	109,690 (22.2)	23,379 (24.1)	12,781 (21)	7,337 (24.9)	915 (24.8)	297 (25)	154,399 (22.5)
≥10	195,800 (39.6)	19,478 (20.1)	8,189 (13.5)	6,833 (23.2)	917 (24.9)	272 (22.9)	231,489 (33.7)

Abbreviations: CEPM, Combined Estrogen plus progestin by manufacturer; CEPP, Combined Estrogen plus progestin by physician; MHT, menopausal hormone therapy
Data are expressed as the number (%) .

Table S3. Subgroup analysis for risk of skin cancer according to major variables in tibolone, Korea National Health Insurance Data, from 2002 to 2019.

Tibolone use	HR (95% CI) [a]	P-value
Tibolone only (without non-MHT)		
Period from menopause to inclusion (years)		
5-9	0.87 (0.558-1.358)	0.54
≥10	0.604 (0.284-1.285)	0.19
Total period of use (months)	1 (0.997-1.003)	0.91
Dosage		
1.25 mg	1.761 (0.653-4.748)	0.26
Over 5 mg	0 (0-.)	>0.99
Prescribed specialty		
Non-gynecology	1.314 (0.967-1.785)	0.08
Dosage of tibolone		
Tibolone 1.25 mg vs Non-MHT	1.499 (0.561-4.001)	0.42
Tibolone 2.5 mg vs Non-MHT	0.812 (0.7-0.942)	0.006
Tibolone 5 mg vs Non-MHT	0.001 (0-4.78E+60)	0.93

Abbreviations: CI, confidence interval; HR, hazard ratio; MHT, menopausal hormone therapy /
[a] HRs were adjusted for age group, body mass index, socioeconomic status, region, Charlson comorbidity index, parity, age at menarche, age at menopause, smoking, alcohol, physical exercise, period from menopause to inclusion.
